# Antiviral Essential Oil Components Against SARS-CoV-2 in Pre-procedural Mouth Rinses for Dental Settings During COVID-19: A Computational Study

**DOI:** 10.3389/fchem.2021.642026

**Published:** 2021-03-29

**Authors:** Pradeep Kumar Yadalam, Kalaivani Varatharajan, K. Rajapandian, Priyanka Chopra, Deepavalli Arumuganainar, Thilgavathi Nagarathnam, Honglae Sohn, Thirumurthy Madhavan

**Affiliations:** ^1^ Adhiparashakthi Dental College and Hospital, Melmaruvathur, India; ^2^ Department of Periodontics, SRM Kattankulathur Dental College and Hospital, SRM Institute of Science and Technology, Chennai, India; ^3^ Faculty of Dental Sciences, SGT University, Gurugram, India; ^4^ Ragas Dental College and Hospital, Chennai, India; ^5^ Department of Chemistry and Department of Carbon Materials, Chosun University, Gwangju, South Korea; ^6^ Department of Genetic Engineering, Computational Biology Lab, School of Bioengineering, SRM Institute of Science and Technology, Chennai, India

**Keywords:** COVID-19, SARS-CoV-2, pre-procedural mouth rinse, antiviral, dental, molecular docking, conceptual DFT

## Abstract

COVID-19 mainly spreads through cough or sneeze droplets produced by an infected person. The viral particles are mostly present in the oral cavity. The risk of contracting COVID-19 is high in the dental profession due to the nature of procedures involved that produce aerosols. Along with other measures to limit the risk of infection, pre-procedural mouth rinses are beneficial in reducing the viral particles in the oral cavity. In this study, the antiviral efficacy of essential oil components has been determined specifically against SARS-CoV-2 by molecular docking and conceptual DFT approach. Based on the binding affinities of the components against the receptor binding domain of the S1 glycoprotein, cuminal, carvacrol, myrtanol, and pinocarveol were found to be highly active. The molecular descriptor values obtained through conceptual DFT also indicated the above-mentioned components to be active based on the correlation between the structure and the activity of the compounds. Therefore, pre-procedural mouth rinses with these components included may be specifically suitable for dental procedures during the COVID-19 period.

## Introduction

The outbreak of corona virus disease 2019 (COVID-19) in Wuhan, China, has impacted the world in several ways (Lai et al., 2020). This disease, caused by severe acute respiratory syndrome coronavirus 2 (SARS-CoV-2), has swiftly spread across 202 countries in the world due to its highly contagious nature (Peng et al., 2020b). As per the World Health Organization (WHO) report, there have been about 38 million confirmed cases of COVID-19, including one million deaths all over the world (as on October 16, 2020) (https://covid19.who.int/). And in India alone, there are seven million cases with about 100,000 deaths reported (as on October 12, 2020) (WHO Coronavirus Disease, 2020). Despite undertaking serious measures to contain the disease globally, it is still on the rise with no vaccine or drug to control the same. The virus spreads through direct contact with cough and sneeze droplets from an infected person or by touching contaminated surfaces and further by touching the nose or mouth (Dhand and Li, 2020). Once a person contracts the disease, the viral particles are mostly housed in the nasal cavity, oropharynx, nasopharynx, and salivary secretions (Han and Ivanovski, 2020; Krajewska Wojciechowska et al., 2020). An infected person displays symptoms such as fever, cough, and cold, a there have been reports indicating that asymptomatic carriers also spread the disease (Qu et al., 2020; Yu and Yang, 2020).

**FIGURE 1:**
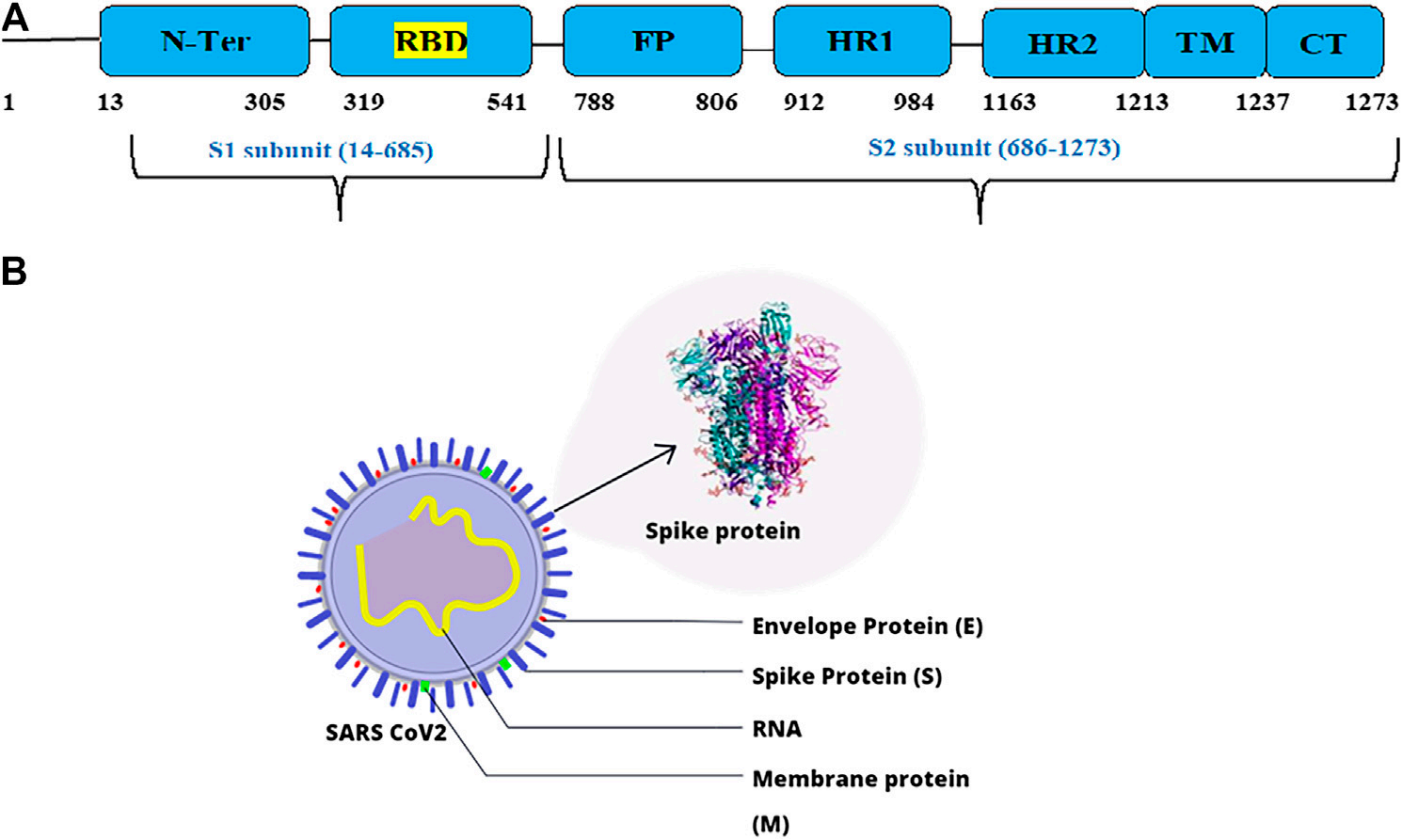
**(A)** Domains of S glycoprotein. **(B)** Structure of SARS-CoV-2.

The nature of dental doctors' work mostly involves being in close proximity with patients and exposure to saliva and blood from aerosols generated from regular dental procedures, which puts them at high risk of viral infection (Li et al., 2020; Meng et al., 2020; Peng et al., 2020a). The droplets may infect the dentist if they are large in size; otherwise, they may remain suspended in the air and cause long-distance transmission in case of smaller droplets (Baghizadeh Fini, 2020). Several studies suggest that SARS-CoV-2 spike protein (1273 amino acid residues) binds to human angiotensin converting enzyme 2 (ACE-2) and utilizes it as a cellular entry receptor for binding and replication (Gurwitz, 2020; Verdecchia et al., 2020; Ziegler et al., 2020). The spike (S) protein is composed of two subunits, namely, S1 and S2. The receptor binding domain (RBD) of the S1 protein (319–541 residues) binds to the ACE-2 cell receptor, followed by fusion, which involves the S2 protein. The RBD lies in the C-terminal domain of the S1 protein, which has more residues that directly interact with the ACE-2 receptor when compared to the N-terminal domain (Huang et al., 2020). The domains of S glycoprotein and structure of SARS-CoV-2 are depicted in Figure 1. Hence, this region is a critical target for antibodies or antiviral compounds. ACE-2 receptors are abundantly present in the salivary glands and lungs (Xu et al., 2020). Therefore, dental professionals must exercise extreme care in terms of safety to prevent nosocomial infection. Dental societies and associations have laid down guidelines to control the transmission of the disease by suggesting dental professionals either completely stop providing dental services or postpone elective treatments and provide primary care through telemedicine services. Only emergency treatments are permitted to be performed by wearing personal protective equipment (PPE) and treating the patients with pre-procedural mouth rinse (PPMR) as a precaution to avoid any possible infection (Jevon and Shamsi, 2020; Nimbulkar et al., 2020). Recent studies have acknowledged the effectiveness of PPMR components such as povidone-iodine, 0.12%-chlorhexidine gluconate, cetylpyridinium chloride, chloroxylenol, benzalkonium chloride, and cetrimide/chlorhexidine in dental care to limit the viral load prior to treatment (Herrera et al., 2020; Meng et al., 2020). Certain essential oil (EO) components such as menthol, thymol, eugenol, and eucalyptol are common active ingredients in mouth rinses (Vlachojannis et al., 2013; Alshehri, 2018). Essential oils are a complex mixture of aromatic compounds that are known for antimicrobial activity against a host of microbes (Bakkali et al., 2008). The activity of these compounds is mostly related to their structure. Previously, numerous studies have proven the efficacy of EOs against many viruses such as herpes simplex virus (type 1 and type 2), influenza virus adenovirus type 3, and poliovirus (Minami et al., 2003; Koch et al., 2008; Swamy et al., 2016; Tariq et al., 2019). The study of synergistic activity among the EO components may lead to better antimicrobial activity. The main advantage of using EOs for therapy, against synthetic drugs, is that they fall under the GRAS (generally regarded as safe) category, whereas synthetic drugs have to undergo various levels of safety and toxicity testing, which is time-consuming. EOs are generally used for therapeutic benefits in complementary and alternative medicine (CAM) to treat infectious, non-infectious, and psychological disorders. Hence, in this study, we aim to identify EO components that are comparable or better in terms of activity, in comparison with the ones that are commonly used.

*In silico* techniques such as molecular docking and conceptual DFT have been employed in this study. The EO components have been docked to the RBD of the spike glycoprotein (S1) since this protein is a key target for many inhibitors because of its involvement in ACE-2 binding. The major objectives of this study are to determine the best set of inhibitors of spike protein based on the binding affinity calculations and to assess the activity of the top inhibitors based on their structure–activity relationship obtained by conceptual DFT calculations.

## Materials and Methods

### Selection and Preparation of Protein Structure

The target protein considered for this study is the RBD of the SARS-CoV-2 S1 subunit, since it is primarily involved in interaction with ACE-2. The 3D structure of this protein possessing PDB ID 6M0J was retrieved from the Protein Data Bank (http://www.rcsb.org/). Initial preparation of the protein structure involved removal of water molecules and co-crystal ligands such as NAG, Cl, Zn, and ACE-2 structure which was bound to the RBD using PyMol software (http://www.pymol.org/). The protein was further prepared for docking by adding charges, energy minimization, fixing side chains and atom bumps, and using PyRx virtual screening software. Subsequently, the protein was converted to the PDBQT file format to render it readable by AutoDock Vina in PyRx software (Trott and Olson, 2010; Dallakyan and Olson, 2015).

### Selection and Preparation of Ligands

The ligands chosen for this study are the components of certain EOs which are known to possess high antimicrobial activity against a broad range of microorganisms. Thymol, eucalyptol, menthol, and eugenol are widely used in most of the pre-procedural mouth rinses used by dentists (Baptista-Silva et al., 2020). These components are majorly present in thyme, eucalyptus, and clove essential oils. Therefore, other essential oil components are chosen along with these standard compounds for comparison purposes.

The 3D structure of the ligands was obtained from the PUBCHEM database (https://pubchem.ncbi.nlm.nih.gov/) in the SDF (structure data file) format. PUBCHEM is a database maintained by the NCBI, which consists of chemical and structure information of compounds that can be freely downloaded along with descriptive datasets. The ligand molecules were imported to the PyRx software using OpenBabel control (O’Boyle et al., 2011). They were prepared by adding charges and optimized using the universal force field (UFF). Furthermore, the ligands were also converted to the PDBQT format, as required by AutoDock Vina. The 2D images of the ligands are presented in Table 1.

**TABLE 1:** 2D structures of the ligands (EO components).

Compound name	Structure	Compound name	Structure
α-Terpinene	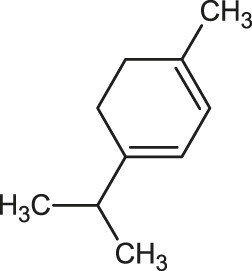	Carvacrol	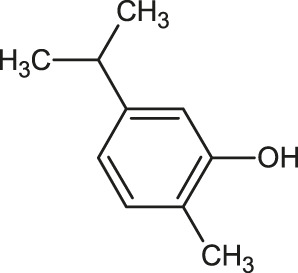
Anethole	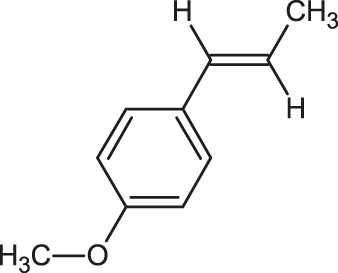	Caryophyllene	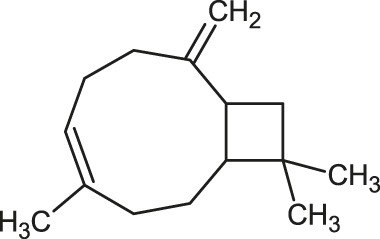
Camphene	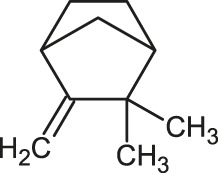	Cinnamaldehyde	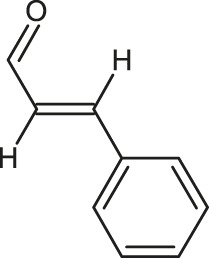
Cinnamyl acetate	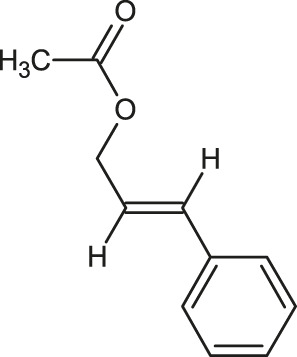	Citronellol	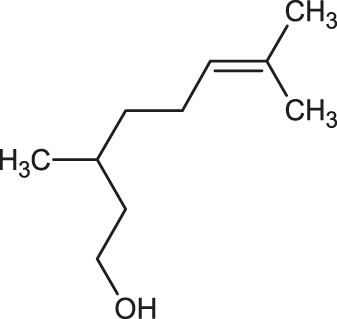
Citral	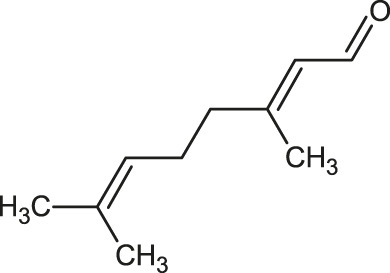	Cuminal	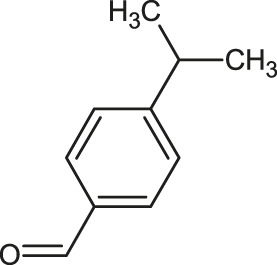
Citronellal	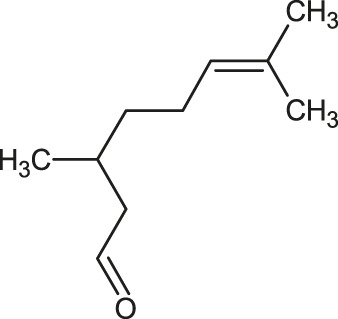	Estragole	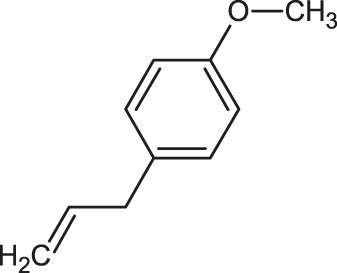
Eucalyptol	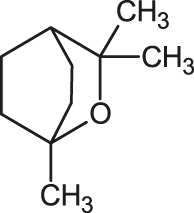	Limonene	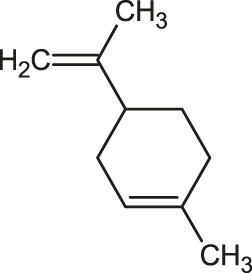
Eugenol	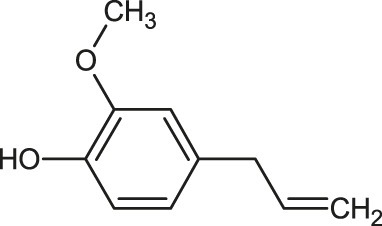	Linalool	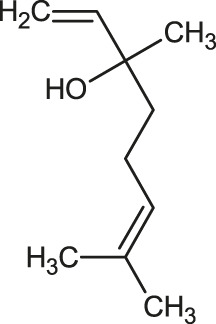
Fenchol	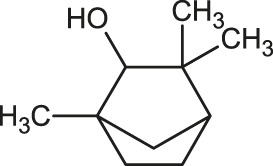	Menthol	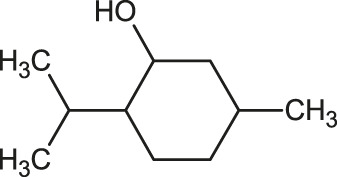
Geraniol	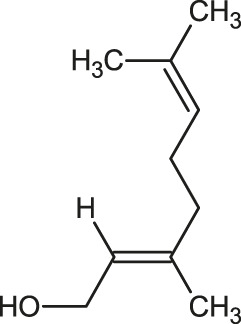	Myrtanol	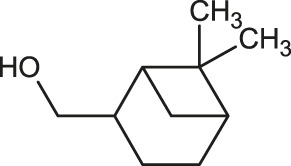
Ocimene	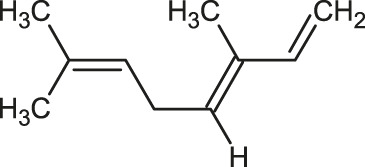	Sabinene	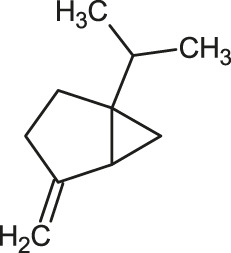
*p*-Cymene	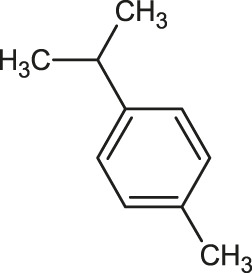	Sylvestrene	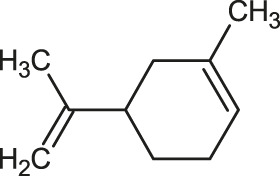
Pinocarveol	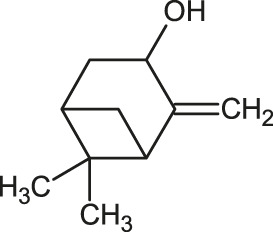	Terpinen-4-ol	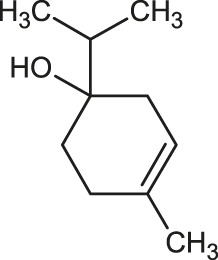
Pulegone	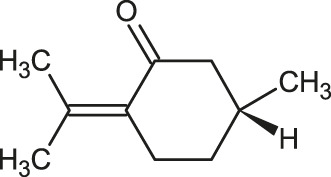	Thujene	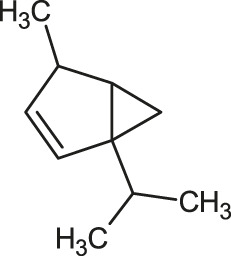
Thymol	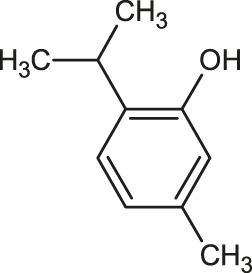	Zingiberene	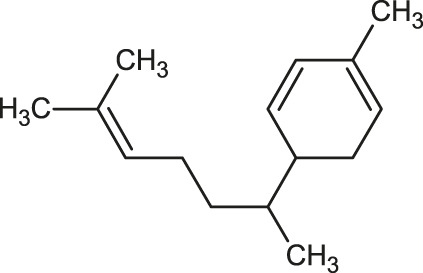

### Binding Site Selection and Molecular Docking

Prior to docking, selection of the appropriate binding site for the ligands is of paramount importance for deriving reliable inference from docking results. One particular binding site has been well characterized by Choudhary et al., 2020; Kulkarni et al., 2020; and Prajapat et al., 2020. Therefore, the site on the RBD with residues Tyr453, Arg454, Leu455, Lys458, Ser459, Ser469, Glu471, Pro491, Leu492, Gln493, and Tyr489 was chosen for docking of ligands. This site of the RBD of S1 protein is also involved in binding to ACE-2.

Molecular docking is performed *in silico* to assess the affinity of binding between a macromolecule and a set of small molecules based on the scores generated by the software for every interaction. In this study, docking was performed using AutoDock Vina in PyRx virtual screening open source software. AutoDock Vina is an upgraded version of AutoDock 4.0 in terms of speed and accuracy of binding mode prediction. In the PyRx software, the protein and ligand molecules to be docked are selected under the Vina Wizard control. The grid which appears on the protein is modified in dimensions according to the area around the binding site. The “Run Vina” control is selected to start the docking process. The results can be viewed under the “Analyze Results” tab and can also be exported in the CSV format to the working directory.

### Conceptual DFT

Conceptual DFT (CDFT) is a subfield of DFT (density functional theory). This technique has been employed in this study to observe the chemical behavior of a molecule based on the electron density in the molecular orbitals (Geerlings and de Proft, 2008). DFT and CDFT are mainly based on the Hohenberg–Kohn theorem (Hohenberg and Kohn, 1964). About 10 different molecular descriptors are calculated as a part of the CDFT study that defines the molecular activity of the components. They are the total energy, lowest unoccupied molecular orbital (LUMO), highest occupied molecular orbital (HOMO), energy gap (ΔE), global softness (σ), absolute hardness (η), molecular dipole moment, electronegativity (χ), electrophilicity index (ω), and chemical potential (μ). These descriptors can provide prominent insights into the structure–activity relationship of molecules.

## Results

### Molecular Docking

**FIGURE 2:**
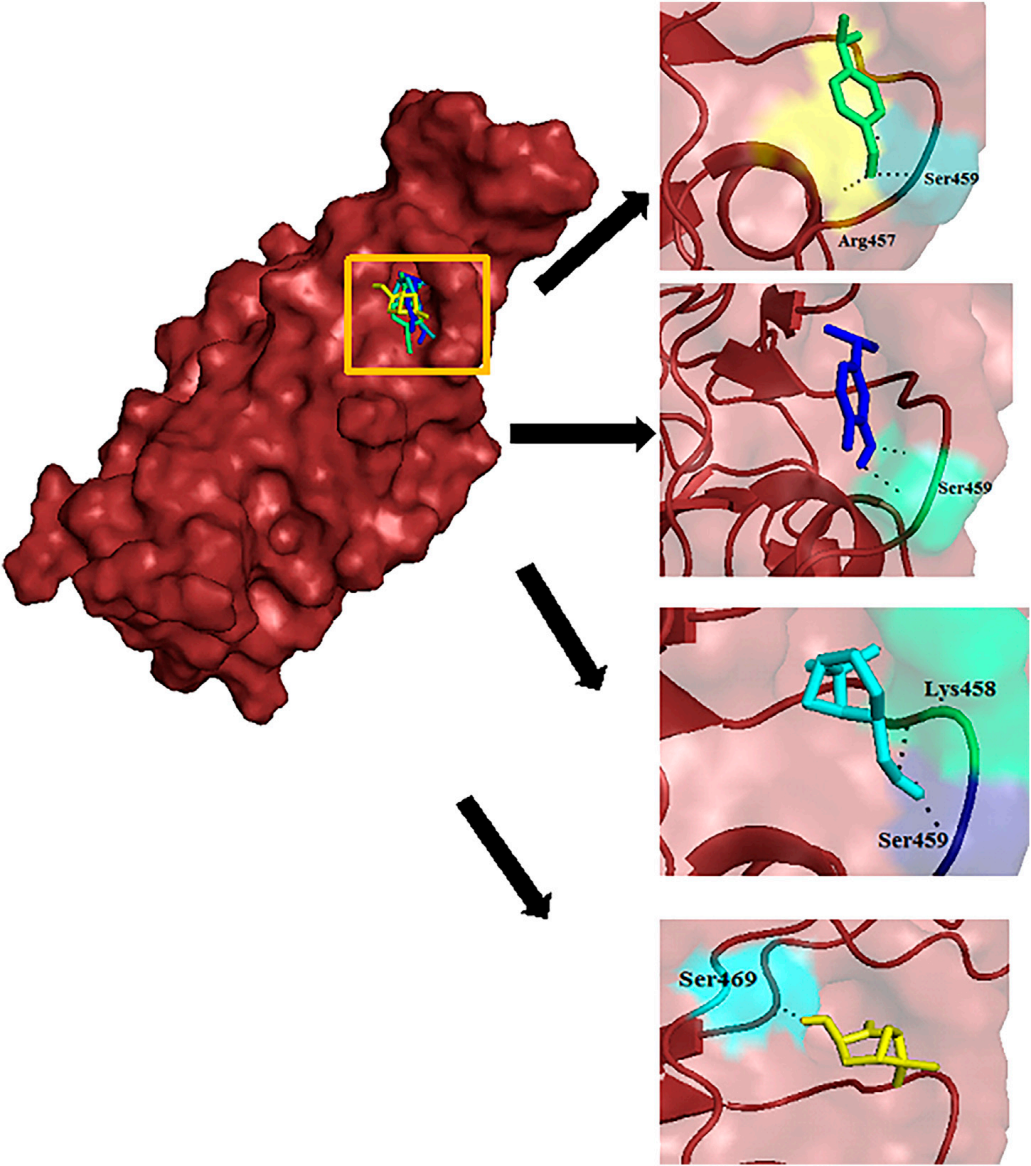
Docked poses of cuminal (green), carvacrol (blue), myrtanol (teal), and pinocarveol (yellow) in the binding site of the RBD of S glycoprotein. The hydrogen bonding between ligands and amino acid residues is depicted.

**FIGURE 3:**
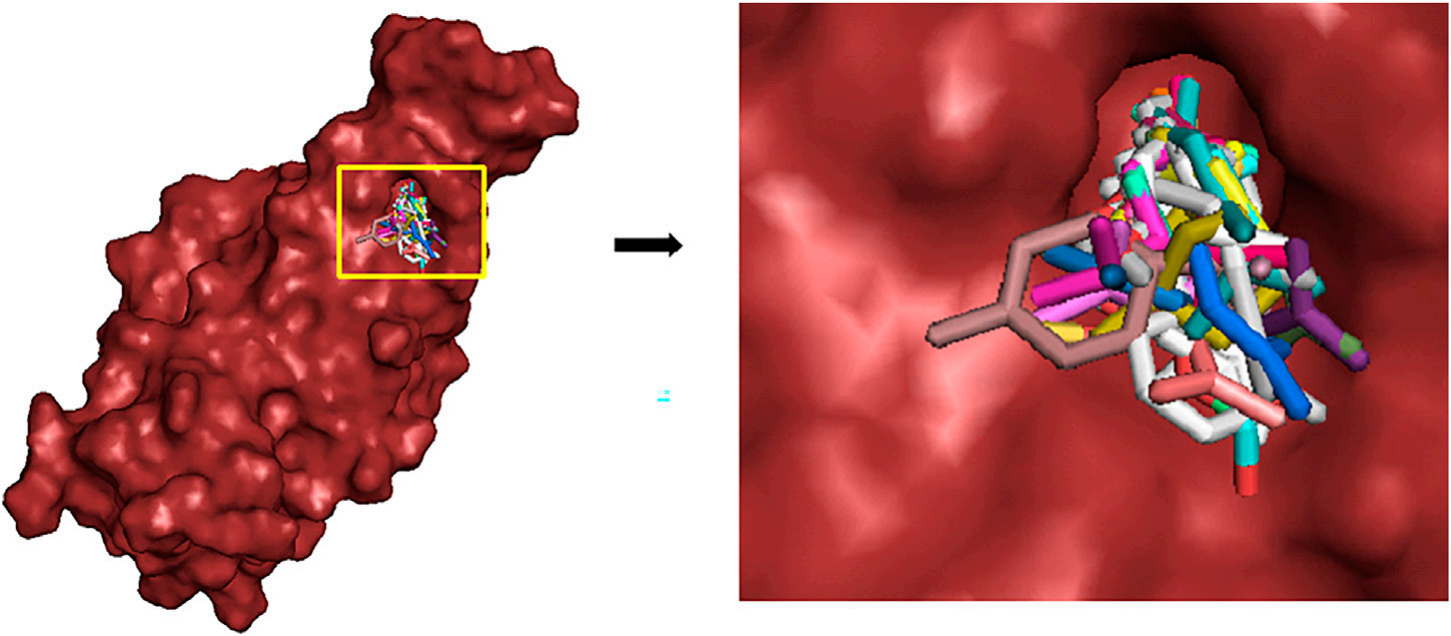
Ligands docked in the binding site of the RBD of S protein.

Docking technique essentially aids in identifying the best inhibitors to a particular protein based on the binding affinity scores generated for various conformations of the docked poses. Visualization tools such as PyMol further help in locating the ligands in the binding pocket along with the bonds exhibited with the neighboring residues. In this case, all 30 EO components were docked in the binding site specified during the docking run. Among them, carvacrol, cuminal, myrtanol, and pinocarveol displayed the best binding affinity with the spike protein with scores of −4.9 kcal/mol, −4.9 kcal/mol, −5.3 kcal/mol, and 5.0 kcal/mol, respectively, and they formed hydrogen bonding with residues Ser459, Arg457, Ser469, and Lys458. Thymol, eugenol, eucalyptol, and menthol, which were also docked for comparison purposes, scored −5.4 kcal/mol, −4.9 kcal/mol, −4.2 kcal/mol, and 5.0 kcal/mol, respectively. Zingiberene and sylvestrene too displayed good binding affinity with scores of −5.2 kcal/mol and −5.1 kcal/mol, respectively, but these components did not make any hydrogen bonds with the residues in vicinity. The stability of all the ligands in the pocket may be attributed to numerous hydrophobic residues present around the site. The docking scores along with hydrogen bonding and hydrophobic interaction information are tabulated in Table 2. It is clear from this study that the components proposed as top inhibitors have displayed almost similar or better activity when compared to the EO components used in conventional PPMRs. Figure 2 illustrates the docked poses of selected inhibitors of SARS-CoV-2 with the hydrogen bonding made by them with the residues. Figure 3 illustrates the docked poses of all the 30 ligands in the binding pocket of the RBD of S protein.

**TABLE 2:** Binding affinities of the EO components with the RBD of S protein along with the H-bond and hydrophobic interactions made with the amino acid residues. EO components with better binding affinities are represented in bold.

Compound name	Binding affinity (kcal/mol)	H-bond interactions	Hydrophobic interactions
α-Terpinene	−4.3	—	Tyr449, Tyr451, Tyr453, Leu455, Phe456, Leu461, Ile468, Thr470, Ile472
Anethole	−4.8	—	
Camphene	−4.4	—	
**Carvacrol**	**−4.9**	Ser459	
Caryophyllene	−4.7	—	
Cinnamaldehyde	−4.6	Tyr473	
Cinnamyl acetate	−4.7	Arg454	
Citral	−4.0	Ser459	
Citronellal	−4.4	Ser459	
Citronellol	−4.4	Arg454	
Cuminal	**−4.9**	Arg457, Ser459	
Estragole	−4.7	Arg457	
Eucalyptol	−4.2	Lys458	
Eugenol	**−4.9**	Arg457, Phe456	
Fenchol	−4.6	—	
Geraniol	−4.6	Arg454, Phe456	
Limonene	−4.6	—	
Linalool	−4.7	Asp467, Ser469	
Menthol	**−5.0**	—	
Myrtanol	**−5.3**	Ser459, Lys458	
Ocimene	−4.0	—	
p-Cymene	−4.8	—	
Pinocarveol	**−5.0**	Ser469	
Pulegone	−4.8	Ser459	
Sabinene	−4.3	—	
Sylvestrene	**−5.1**	—	
Terpinen-4-ol	−4.8	Arg457, Asp467	
Thujene	−4.8	—	
Thymol	**−5.4**	Arg457, Phe456	
Zingiberene	**−5.2**	—	

### Conceptual DFT

**FIGURE 4:**
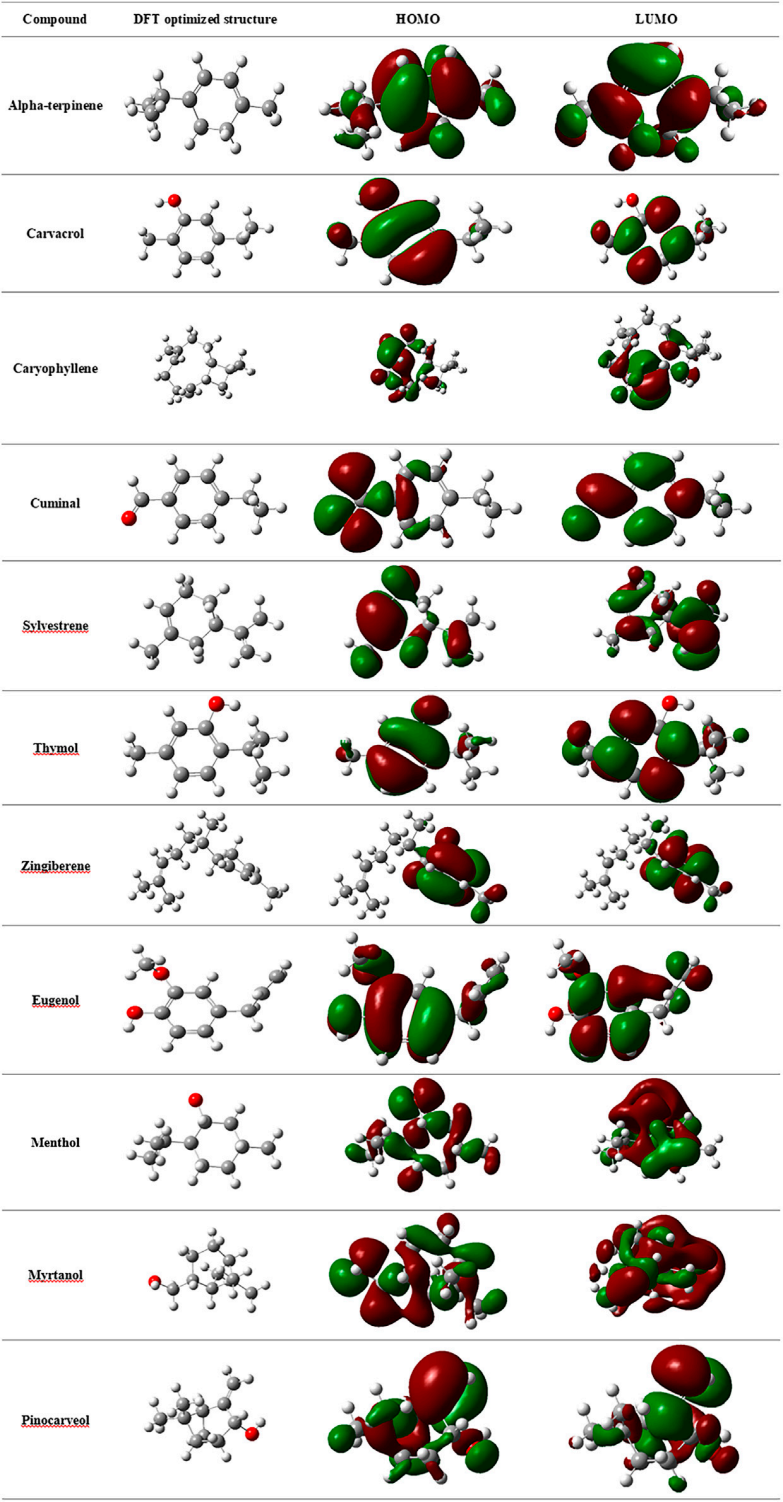
Electron density maps of the HOMO and LUMO of selected essential oil components.

Optimization of the EO components was performed using Gaussian 16 (Frisch et al., 2016) with B3LYP function (Becke, 1988) and 6-31G(d) basis set. The energies of the molecular orbitals represented as HOMO (*E*_*HOMO*_) and LUMO (*E*_*LUMO*_) were calculated on the basis of Fukui’s theory (Fukui, 1982). The values of each of the descriptors were derived for the selected EO components. The HOMO and LUMO represent the ability of the compounds in donating and accepting electrons, respectively. The energy gap (*ΔE*) is the difference in energies between two molecules orbitals, which is given by *ΔE = E*_*LUMO*_–*E*_*HOMO*_. *ΔE* essentially represents the energy needed to perform transition of molecules from the HOMO to the LUMO, and hence, it is directly proportional to the molecular reactivity (Mert et al., 2011). In this study, larger *ΔE* values were attributed to a wide range of *E*_*LUMO*_ values. Table 3 provides the statistics of DFT‐based molecular descriptors of selected EO components. It can be observed from the table that cuminal showed the lowest *ΔE*, whereas zingiberene displayed the largest *ΔE*. It is understood that the lower the energy gap, the higher the activity of the molecules, which can be correlated with the transition of molecules from the HOMO to the LUMO. Carvacrol, caryophyllene, pinocarveol, and sylvestrene also exhibited low *ΔE*. The electron density maps depicting the density of electrons in different regions of the molecules are presented in Figure 4. Mert et al., 2011, have pointed out that the molecular dipole moment of a molecule is directly proportional to its chemical reactivity. Cuminal has the highest dipole moment with 3.84 debye, followed by pinocarveol with 1.70 debye and myrtanol with 1.64 debye, which is higher than that of eugenol, menthol, and thymol. Carvacrol and thymol scored 1.45 debye. Electronegativity of a compound is an index of the ability of a molecule to accept electrons. It is an important indicator of efficiency of inhibition of the molecule. The lower the electronegativity of a molecule is, the higher its efficiency of inhibition will be. Cuminal has the lowest electronegativity index (–4.21). This index for other components was almost in the range of –3.15 to –2.82. The results of conceptual DFT are in agreement with the docking results.

**TABLE 3:** Statistics of DFT based molecular descriptors of selected EO components.

Compound	Total energy (E γ) (in eV)	Molecular dipole moment (debye)	E_HOMO_	E_LUMO_	HOMO/LUMO gap (ΔE)	Absolute hardness (η)	Global softness (σ)	Electronegativity (χ)	Chemical potential (μ)	Electrophilicity index (ω)
α-Terpinene	−10631.09	0.49	−5.23	−0.26	4.97	2.49	0.20	−2.75	2.75	1.52
Carvacrol	−12645.87	1.45	−5.75	0.19	5.94	2.97	0.17	−2.78	2.78	1.30
Caryophyllene	−15945.58	0.35	−5.95	0.53	6.48	3.24	0.15	−2.71	2.71	1.13
Cuminal	−12612.99	3.84	−6.83	−1.59	5.24	2.62	0.19	−4.21	4.21	3.39
Eugenol	−14658.45	1.50	−5.72	0.08	5.80	2.90	0.17	−2.82	2.82	1.37
Menthol	−12744.29	1.50	−6.90	2.01	8.91	4.45	0.11	−2.44	2.44	0.67
Myrtanol	−12710.09	1.64	−6.91	1.89	8.80	4.40	0.11	−2.51	2.51	0.72
Pinocarveol	−12676.80	1.70	−6.88	−1.50	5.38	3.48	0.14	−3.15	3.15	2.71
Sylvestrene	−10630.83	0.35	−6.13	0.77	6.91	3.45	0.14	−2.68	2.68	1.04
Thymol	−12645.82	1.45	−5.72	0.18	5.90	2.95	0.17	−2.77	2.77	1.30
Zingiberene	−15835.79	0.37	−7.96	3.89	11.86	5.93	0.08	−2.04	2.04	0.35

## Discussion

Due to the sudden outbreak of COVID-19, the standard procedures of operation had to be modified in almost every sector, especially in the field of medicine and dentistry since they involve frontline care givers. Dental professionals have to exercise extra caution because of the high risk of nosocomial infection through aerosol-generating procedures. Although physical protection from the virus by wearing safety gear such as PPE is recommended, an effective antiviral PPMR may ensure safety even in case the patient is infected but asymptomatic. Recent literature suggests numerous mouth rinses that can effectively reduce the viral load in the oral cavity. Povidone-iodine (PVP-I) oral rinse has been found to be effective in various studies conducted by Tessema et al., 2020; Bidra et al., 2020; and Pelletier et al., 2021. Mouth rinses containing 1% PVP-I exhibited a virucidal activity higher than 99.99%, which corresponds to a reduction of viral load greater than 4 log_10_. The use of PVP-I has been contraindicated in patients with an allergy to iodine, thyroid disease, and pregnancy. Chlorhexidine (CHX) is a broad-spectrum antiseptic that has long been known to be effective against herpes simplex virus (HSV), human immunodeficiency virus (HIV), and hepatitis B virus (HBV) (Brookes et al., 2020). The effectiveness of CHX specifically against SARS-CoV-2 has not been well established yet. In comparison with PVP-I, hydrogen peroxide (H_2_O_2_) has been found to be less effective by a study conducted by Ather et al., 2020. Certain essential oil (EO) components such as thymol, eugenol, menthol, methyl salicylate, and eucalyptol are common major ingredients in mouth rinses recommended by the American Dental Association (ADA) (Alshehri, 2018). The activity of these components has been well established by several studies against a wide array of microbes, including viruses. The main aim of this study was to explore other components with comparable or better activity than the existing ones by *in silico* methods. Moreover, EO components are safe since they fall under the generally regarded as safe (GRAS) category. Cuminal, myrtanol, carvacrol, caryophyllene, pinocarveol, and sylvestrene were found to have inhibitory effects against SARS-CoV-2. A number of recent *in silico* studies have predicted the anitiviral activity of EO components against SARS-CoV-2. Kulkarni et al., 2020, performed a similar study with the same target protein and found that cinnamaldehyde, anethole, thymol, and carvacrol were highly active. Similar results were obtained by Asif et al., 2020, and Senthil Kumar et al., 2020. Boukhatem, 2020, have discussed how EOs could have an inhibitory effect on SARS-CoV-2, similar to the effect they have had on other viruses. Thuy et al., 2020, predicted that 17 compounds of garlic oil interacted with the viral main protease (Mpro) of SARS-CoV-2. Da Silva et al., 2020, predicted that (E,E)-α-farnesene, (E,E)-farnesol, and (E)-nerolidol interacted with SARS-CoV-2 Mpro, thereby inhibiting viral replication, out of 171 EO components. So far, no *in vitro* or *in vivo* studies have established the efficacy of these compounds. This study has resulted in predicting EO components that can increase the efficiency of conventional PPMRs by reducing the viral load in the oropharyngeal cavity, specifically against SARS-CoV-2.

## Conclusion

Providing dental care treatment to patients, while reducing the risk of highly contagious viral infection caused by SARS-CoV-2 is a challenge for dental professionals. Through this study, we conclude that EO components such as cuminal, carvacrol, myrtanol, caryophyllene, pinocarveol, and sylvestrene are good inhibitors of the S1 glycoprotein of coronavirus by *in silico* methods. Hence, these components can be proposed to be effective antiviral ingredients of pre-procedural mouth rinses recommended to be administered to patients for effective reduction of viral load in the oropharyngeal cavity. The futurology of this study indicates *in vitro* and *in vivo* testing of the same to confirm the antiviral efficiency of the proposed EO components, specifically against SARS-CoV-2.

## Data Availability

The authors acknowledge that the data presented in this study must be deposited and made publicly available in an acceptable repository, prior to publication.

## References

[B1] Alshehri F. A. (2018). The use of mouthwash containing essential oils (LISTERINE®) to improve oral health: a systematic review. Saudi Dent. J. 30, 2–6. 10.1016/j.sdentj.2017.12.004 30166864 PMC6112363

[B2] Asif M. Saleem M. Saadullah M. Yaseen H. S. al Zarzour R. (2020). COVID-19 and therapy with essential oils having antiviral, anti-inflammatory, and immunomodulatory properties. Inflammopharmacology 28, 1153–1161. 10.1007/s10787-020-00744-0 32803479 PMC7427755

[B3] Ather A. Patel B. Ruparel N. B. Diogenes A. Hargreaves K. M. (2020). Coronavirus disease 19 (COVID-19): implications for clinical dental care. J. Endod. 46, 584–595. 10.1016/j.joen.2020.03.008 32273156 PMC7270628

[B4] Baghizadeh Fini M. (2020). What dentists need to know about COVID-19. Oral Oncol. 105, 104741. 10.1016/j.oraloncology.2020.104741 32380453 PMC7186204

[B5] Bakkali F. Averbeck S. Averbeck D. Idaomar M. (2008). Biological effects of essential oils-a review. Food Chem. Toxicol. 46, 446–475. 10.1016/j.fct.2007.09.106 17996351

[B6] Baptista-Silva S. Borges S. Ramos O. L. Pintado M. Sarmento B. (2020). The progress of essential oils as potential therapeutic agents: a review. J. Essent. Oil Res. 32, 279–295. 10.1080/10412905.2020.1746698

[B7] Becke A. D. (1988). Density-functional exchange-energy approximation with correct asymptotic behavior. Phys. Rev. A. Gen. Phys. 38, 3098–3100. 10.1103/PhysRevA.38.3098 9900728

[B8] Bidra A. S. Pelletier J. S. Westover J. B. Frank S. Brown S. M. Tessema B. (2020). Rapid in-vitro inactivation of severe acute respiratory syndrome coronavirus 2 (SARS-CoV-2) using povidone-iodine oral antiseptic rinse. J. Prosthodont. 29, 529–533. 10.1111/jopr.13209 32511851 PMC7300649

[B9] Boukhatem M. N. (2020). Effective antiviral activity of essential oils and their characteristics terpenes against coronaviruses: an update. J. Pharmacol. Clin. Toxicol. 8, 1–8.

[B10] Brookes Z. L. S. Bescos R. Belfield L. A. Ali K. Roberts A. (2020). Current uses of chlorhexidine for management of oral disease: a narrative review. J. Dent. 103, 103497. 10.1016/j.jdent.2020.103497 33075450 PMC7567658

[B11] Choudhary S. Malik Y. S. Tomar S. (2020). Identification of SARS-CoV-2 cell entry inhibitors by drug repurposing using in silico structure-based virtual screening approach. Front. Immunol. 11, 1664. 10.3389/fimmu.2020.01664 32754161 PMC7365927

[B12] Da Silva J. K. R. Figueiredo P. L. B. Byler K. G. Setzer W. N. (2020). Essential oils as antiviral agents. Potential of essential oils to treat SARS-CoV-2 infection: an in-silico investigation. Int. J. Mol. Sci. 21, 1–37. 10.3390/ijms21103426 PMC7279430 32408699

[B13] Dallakyan S. Olson A. J. (2015). Small-molecule library screening by docking with PyRx. Methods Mol. Biol. 1263, 243–250. 10.1007/978-1-4939-2269-7_19 25618350

[B14] Dhand R. Li J. (2020). Coughs and sneezes: their role in transmission of respiratory viral infections, including SARS-CoV-2. Am. J. Respir. Crit. Care Med. 202, 651–659. 10.1164/rccm.202004-1263PP 32543913 PMC7462404

[B15] Frisch M. J. Trucks G. W. Schlegel H. B. Scuseria G. E. Robb M. A. Cheeseman J. R. (2016). Gaussian 16, Wallingford, CT: Gaussian Inc.

[B16] Fukui K. (1982). The role of frontier orbitals in chemical reactions (nobel lecture). Angew. Chem. Int. Ed. Engl. 21, 801–809. 10.1002/anie.198208013

[B17] Geerlings P. de Proft F. (2008). Conceptual DFT: the chemical relevance of higher response functions. Phys. Chem. Chem. Phys. 10, 3028–3042. 10.1039/b717671f 18688366

[B18] Gurwitz D. (2020). Angiotensin receptor blockers as tentative SARS-CoV-2 therapeutics. Drug Dev. Res. 81, 537–540. 10.1002/ddr.21656 32129518 PMC7228359

[B19] Han P. Ivanovski S. (2020). Saliva-friend and foe in the COVID-19 outbreak. Diagnostics 10, 290. 10.3390/diagnostics10050290 PMC7277967 32397487

[B20] Herrera D. Serrano J. Roldán S. Sanz M. (2020). Is the oral cavity relevant in SARS-CoV-2 pandemic? Clin. Oral Investig. 24, 2925–2930. 10.1007/s00784-020-03413-2 PMC7309196 32577830

[B21] Hohenberg P. Kohn W. (1964). Inhomogeneous electron gas. Phys. Rev. 136, B864. 10.1103/PhysRev.136.B864

[B22] Huang Y. Yang C. Xu X. F. Xu W. Liu S. W. (2020). Structural and functional properties of SARS-CoV-2 spike protein: potential antivirus drug development for COVID-19. Acta Pharmacol. Sin. 41, 1141–1149. 10.1038/s41401-020-0485-4 32747721 PMC7396720

[B23] Jevon P. Shamsi S. (2020). COVID-19 and medical emergencies in the dental practice. Br. Dent J. 229, 19–24. 10.1038/s41415-020-1782-5 32651513 PMC7348561

[B24] Koch C. Reichling J. Schneele J. Schnitzler P. (2008). Inhibitory effect of essential oils against herpes simplex virus type 2. Phytomedicine 15, 71–78. 10.1016/j.phymed.2007.09.003 17976968

[B25] Krajewska Wojciechowska J. Krajewski W. Zub K. Zatoński T. (2020). Review of practical recommendations for otolaryngologists and head and neck surgeons during the COVID-19 pandemic. Auris Nasus Larynx 47, 544–558. 10.1016/j.anl.2020.05.022 32540054 PMC7275141

[B26] Kulkarni S. A. Nagarajan S. K. Ramesh V. Palaniyandi V. Selvam S. P. Madhavan T. (2020). Computational evaluation of major components from plant essential oils as potent inhibitors of SARS-CoV-2 spike protein. J. Mol. Struc. 1221, 128823. 10.1016/j.molstruc.2020.128823 PMC7334662 32834111

[B27] Lai C. C. Shih T. P. Ko W. C. Tang H. J. Hsueh P. R. (2020). Severe acute respiratory syndrome coronavirus 2 (SARS-CoV-2) and coronavirus disease-2019 (COVID-19): the epidemic and the challenges. Int. J. Antimicrob. Agents 55, 105924. 10.1016/j.ijantimicag.2020.105924 32081636 PMC7127800

[B28] Li Y. Ren B. Peng X. Hu T. Li J. Gong T. (2020). Saliva is a non-negligible factor in the spread of COVID-19. Mol. Oral Microbiol. 35, 141–145. 10.1111/omi.12289 32367576 PMC7267240

[B29] Meng L. Hua F. Bian Z. (2020). Coronavirus disease 2019 (COVID-19): emerging and future challenges for dental and oral medicine. J. Dent. Res. 99, 481–487. 10.1177/0022034520914246 32162995 PMC7140973

[B30] Mert B. D. Erman Mert M. Kardaş G. Yazici B. (2011). Experimental and theoretical investigation of 3-amino-1,2,4-triazole-5-thiol as a corrosion inhibitor for carbon steel in HCl medium. Corros. Sci. 53, 4265–4272. 10.1016/j.corsci.2011.08.038

[B31] Minami M. Kita M. Nakaya T. Yamamoto T. Kuriyama H. Imanishi J. (2003). The inhibitory effect of essential oils on herpes simplex virus type-1 replication *in vitro* . Microbiol. Immunol. 47, 681–684. 10.1111/j.1348-0421.2003.tb03431.x 14584615

[B32] Nimbulkar G. Dubey N. Mandwar S. Dharmapuria S. Reche A. Chhabra K. G. (2020). Dental practice guidelines in the precariousness of COVID-19: a review. Int. J. Curr. Res. Rev. 12, 82–87. 10.31782/IJCRR.2020.12195

[B33] O’Boyle N. M. Banck M. James C. A. Morley C. Vandermeersch T. Hutchison G. R. (2011). Open babel: an open chemical toolbox. J. Cheminform. 3, 33. 10.1186/1758-2946-3-33 21982300 PMC3198950

[B34] Pelletier J. S. Tessema B. Frank S. Westover J. B. Brown S. M. Capriotti J. A. (2021). Efficacy of povidone-iodine nasal and oral antiseptic preparations against severe Acute Respiratory syndrome-coronavirus 2 (SARS-CoV-2). Ear Nose Throat J. 100, 192S–196S. 10.1177/0145561320957237 32951446

[B35] Peng X. Xu X. Li Y. Cheng L. Zhou X. Ren B. (2020a). Transmission routes of 2019-nCoV and controls in dental practice. Int. J. Oral Sci. 12, 9. 10.1038/s41368-020-0075-9 32127517 PMC7054527

[B36] Peng Y. Li C. Rong Y. Chen X. Chen H. (2020b). Retrospective analysis of the accuracy of predicting the alert level of COVID-19 in 202 countries using Google Trends and machine learning. J. Glob. Health 10, 020511. 10.7189/jogh.10.020511 33110594 PMC7567446

[B37] Prajapat M. Shekhar N. Sarma P. Avti P. Singh S. Kaur H. (2020). Virtual screening and molecular dynamics study of approved drugs as inhibitors of spike protein S1 domain and ACE2 interaction in SARS-CoV-2. J. Mol. Graph. Model. 101, 107716. 10.1016/j.jmgm.2020.107716 32866780 PMC7442136

[B38] Qu J. Chang L. K. Tang X. Du Y. Yang X. Liu X. (2020). Clinical characteristics of COVID-19 and its comparison with influenza pneumonia. Acta Clinica Belgica: Int. J. Clin. Lab. Med. 75, 348–356. 10.1080/17843286.2020.1798668 32723027

[B39] Senthil Kumar K. J. Vani M. G. Wang C. S. Chen C. C. Chen Y. C. Lu L. P. (2020). Geranium and lemon essential oils and their active compounds downregulate angiotensin-converting enzyme 2 (ACE2), a SARS-CoV-2 spike receptor-binding domain, in epithelial cells. Plants 9, 1–12. 10.3390/plants9060770 PMC7355681 32575476

[B40] Swamy M. K. Akhtar M. S. Sinniah U. R. (2016). Antimicrobial properties of plant essential oils against human pathogens and their mode of action: an updated review. Evid. Based Complement. Alternat Med. 2016, 3012462. 10.1155/2016/3012462(2016). 28090211 PMC5206475

[B41] Tariq S. Wani S. Rasool W. Shafi K. Bhat M. A. Prabhakar A. (2019). A comprehensive review of the antibacterial, antifungal and antiviral potential of essential oils and their chemical constituents against drug-resistant microbial pathogens. Microb. Pathog. 134, 103580. 10.1016/j.micpath.2019.103580 31195112

[B42] Tessema B. Frank S. Bidra A. (2020). SARS-CoV-2 viral inactivation using low dose povidone-iodine oral rinse-immediate application for the prosthodontic practice. J. Prosthodont. 29, 459. 10.1111/jopr.13207 32542782 PMC7322989

[B43] Thuy B. T. P. My T. T. A. Hai N. T. T. Hieu L. T. Hoa T. T. Thi Phuong Loan H. (2020). Investigation into SARS-CoV-2 resistance of compounds in garlic essential oil. ACS Omega 5, 8312–8320. 10.1021/acsomega.0c00772 32363255 PMC7123907

[B44] Trott O. Olson A. J. (2010). AutoDock Vina: improving the speed and accuracy of docking with a new scoring function, efficient optimization, and multithreading. J. Comput. Chem. 31, 455–461. 10.1002/jcc.21334 19499576 PMC3041641

[B45] Verdecchia P. Cavallini C. Spanevello A. Angeli F. (2020). The pivotal link between ACE2 deficiency and SARS-CoV-2 infection. Eur. J. Int. Med. 76, 14–20. 10.1016/j.ejim.2020.04.037 PMC7167588 32336612

[B46] Vlachojannis C. Winsauer H. Chrubasik S. (2013). Effectiveness and safety of a mouthwash containing essential oil ingredients. Phytother. Res. 27, 685–691. 10.1002/ptr.4762 22761009

[B47] WHO Coronavirus Disease (2020). (COVID-19) dashboard. Available at: https://covid19.who.int/region/searo/country/ (Accessed December 05 2020).

[B48] Xu R. Cui B. Duan X. Zhang P. Zhou X. Yuan Q. (2020). Saliva: potential diagnostic value and transmission of 2019-nCoV. Int. J. Oral Sci. 12, 11. 10.1038/s41368-020-0080-z 32300101 PMC7162686

[B49] Yu X. Yang R. (2020). COVID-19 transmission through asymptomatic carriers is a challenge to containment. Influenza Other Respir. Viruses 14, 474–475. 10.1111/irv.12743 32246886 PMC7228388

[B50] Ziegler C. G. K. Allon S. J. Nyquist S. K. Mbano I. M. Miao V. N. Tzouanas C. N. (2020). SARS-CoV-2 receptor ACE2 is an interferon-stimulated gene in human airway epithelial cells and is detected in specific cell subsets across tissues. Cell 181, 1016–1035.e19. 10.1016/j.cell.2020.04.035 32413319 PMC7252096

